# Supplementary material for: Long Pentraxin-3 Follows and Modulates Bladder Cancer Progression
Source: Cancers (Basel). 2019 Aug 30;11(9):1277. doi: 10.3390/cancers11091277 (PMC6770810; doi:10.3390/cancers11091277)
Supplement: Supplementary file 1 [file cancers-11-01277-s001.pdf]

Supplementary Materials:

Long Pentraxin-3 Follows and Modulates Bladder Cancer Progression

Table S1. List of primers use in qPCR.

Human Gene	Forward Primer	Reverse Primer
**GAPDH**	GAAGGTCGGAGTCAACGGATT	TGACGGTGCCATGGAATTTG
**PTX3**	CATCTCCTTGCGATTCTGTTTTG	CCCATTCCGAGTGCTCCTGA
**PTX3 promoter region 1 (P1) ***	TGTCATAGGCCGGGTGAGG	CCGCCACACCCAGTTAAT
**PTX3 promoter region 2 (P2) ***	TCGAGACCAGCCTGGCTAA	GCAATGGTGCTCCCGCATC
**PTX3 transcription factors binding site region (TFBS) ***	TGGCACTGCGGTAACGGGA	CCTCCAATTAATCTGACTGC
**PTX3 iCG1 ***	GAGCTACATAACCGGCGA	GCCAAGTTGCAGCCCGTTCC
**PTX3 iCG2 ***	GGAACGGGCTGCAACTTGG	GTCCCATTCCGAGTGCTCC
**PTX3 iCG3 ***	GCACTCGGAATGGGACAAGC	CGCGCACGGCCTCGCCAG
**PTX3 iCG4 ***	CTGGCGAGGCCGTGCGCG	CCTCTAGCACCGCGGCCAG
**PTX3 iCG5 ***	GGGCGCGCCCTGGCCGCG	GTGGGAGGTCCCGGCCCC
**PTX3 iCG6 ***	GGGGAAGCTTTCATGGGAAGC	CCGCCTTGTATGGTGTGCCTG
**PTX3 iCG7 ***	CACAGTCTTGAAATAGACGTC	GAAGACGTGGAAATCCCT AAC
**PTX3 iCG8 ***	GGAGCTCTGGTTTCCCGC	CGGCTGACCCTTCGGGAG
**PTX3 iCG9 ***	GCGGGGGTCAGCGGAATTC	GGGGTGGAGCTGGGATCCC
**FGFR1**	GGGCTGGAATACTGCTACAA	GCCAAAGTCTGCTATCTTCATC
**FGFR2**	GGATAACAACACGCCTCTCTT	GCCCAAAGCAACCTTCTC
**FGFR3**	TGGTGTCCTGTGCCTACC	CCGTTGGTCGTCTTCTTGT
**FGFR4**	AACCGCATTGGAGGCATT	TCTACCAGGCAGGTGTATGT
**FGF2**	TGTGTCTATCAAAGGAGTGTG	CCGTAACACATTTAGAAGCCA
**FGF5**	ATGCAAGTGCCAAGTTCAC	TGTATTGCTGAGGCATAGGT
**FGF6**	GAAAGTGGCTATTTGGTGGG	ATTTCCAGCAGGCTGTAGG
**FGF8**	TCATCCGGACCTACCAACTC	AATCTCCGTGAAGACGCAGT
**FGF10**	GGAGAAAGCTATTCTCTTTCACC	ATCTCCAGGATGCTGTACG
**FGF17**	CAACAAGTTTGCCAAGCTC	TACTTCTCACTCTCAGCCC
**FGF18**	GGACATGTGCAGGCTGGGCTA	GTAGAATTCCGTCTCCTTGCCCTT
**NANOG**	ACCTATGCCTGTGATTTGTGG	AAGTGGGTTGTTTGCCTTTG
**OCT-4**	GCAAAGCAGAAACCCTCGT	ACACTCGGACCACATCCTTC
**CD44**	AACACCAAGCCCAGAGGAC	TCCAAATCTTCCACCAAACC
**CD47**	TCCAAGAATGATGCCTTTCA	TACTGCCATAACTGCCCAAA
**ABCB1**	GGGAGCTTAACACCCGACTTA	GCCAAAATCACAAGGGTTAGCTT
**ABCG2**	ACGAACGGATTAACAGGGTCA	CTCCAGACACACCACGGAT

*Primers used for MIRA analysis as described in: Rubino M. et al. Epigenetic regulation of the extrinsic oncosuppressor PTX3 gene in inflammation and cancer. Oncoimmunology. 2017 May 30;6(7): e1333215. doi: 10.1080/2162402X.2017.1333215.

Table S2. Description of patients (age and gender) and tumor samples (tumor type, grading, T and N evaluation, PTX3 staining).

TYPE	AGE	GENDER	GRADING	pT	N	PTX3 Staining
MI	73	M	HG	T3b	N0	−
MI	57	M	HG	T2b	N0	++
MI	78	M	HG	T3a	N0	−
MI	78	M	HG	T3a	N0	++
MI	51	M	HG	T2b	N0	−
MI	61	M	HG	T2	N0	−
MI	71	M	HG	T3	N0	+
MI	56	M	HG	T3a	N0	+
MI	69	M	HG	T4	N0	−
MI	57	M	HG	T3a	N0	+
MI	57	M	HG	T3a	N0	+
MI	86	F	HG	T3b	N0	hetero
MI	68	M	HG	T3a	N2	hetero
MI	76	M	HG	T2b	N0	hetero
MI	61	M	HG	T2b	N0	hetero
MI	74	M	HG	T3a	N0	hetero
MI	73	M	HG	T4b	N0	+
MI	74	M	HG	T3a	N0	hetero
MI	70	F	HG	T3b	N2	+
MI	61	F	HG	T3b	N1	hetero
MI	84	M	HG	T3a	N0	++
MI	81	M	HG	T4	N0	++
MI	76	M	HG	T3a	N1	+
MI	81	F	HG	T3a	N1	hetero
MI	47	F	HG	T4	N0	hetero
MI	85	F	HG	T4	N1	−
MI	81	M	HG	T4	N0	hetero
MI	87	M	HG	T2b	N0	hetero
MI	74	M	HG	T3a	N0	+
MI	72	M	HG	T2	N0	++
MI	79	M	HG	T3a	N1	+
MI	85	M	HG	T4a	N0	+
MI	54	M	HG	T3a	N0	hetero
MI	89	F	HG	T2	ND	−
MI	73	M	HG	T3b	N0	hetero
MI	66	M	HG	T3a	N2	−
MI	82	M	HG	T4	N1	++
MI	79	F	HG	T3a	N0	−
MI	82	F	HG	T3a	N0	hetero
NMI	69	M	HG	T1	ND	hetero
NMI	73	F	HG	Ta	ND	hetero
NMI	65	M	HG	T1	ND	hetero
NMI	85	M	HG	T1	ND	+
NMI	75	F	HG	T1	ND	hetero
NMI	60	M	HG	T1	ND	hetero
NMI	70	F	HG	Ta	ND	+
NMI	69	M	HG	T1	ND	hetero
NMI	62	F	HG	T1	ND	+
NMI	78	M	HG	Ta	ND	+
NMI	80	M	HG	T1	ND	hetero
NMI	53	M	HG	T1	ND	+
NMI	84	M	HG	Ta	ND	+

NMI	56	M	HG	T1	ND	+
NMI	78	M	HG	Tis	ND	hetero
NMI	67	F	HG	T1	ND	++
NMI	66	M	HG	T1	ND	+
PLG	62	M	LG	Ta	ND	+
PLG	66	M	LG	Ta	ND	+
PLG	48	M	LG	Ta	ND	+
PLG	58	F	LG	Ta	ND	hetero
PLG	75	M	LG	Ta	ND	+

MI= muscle invasive, NMI= non muscle invasive, PLG= papillary low grade, HG= high grade, LG= low grade, hetero= heterogeneous staining)

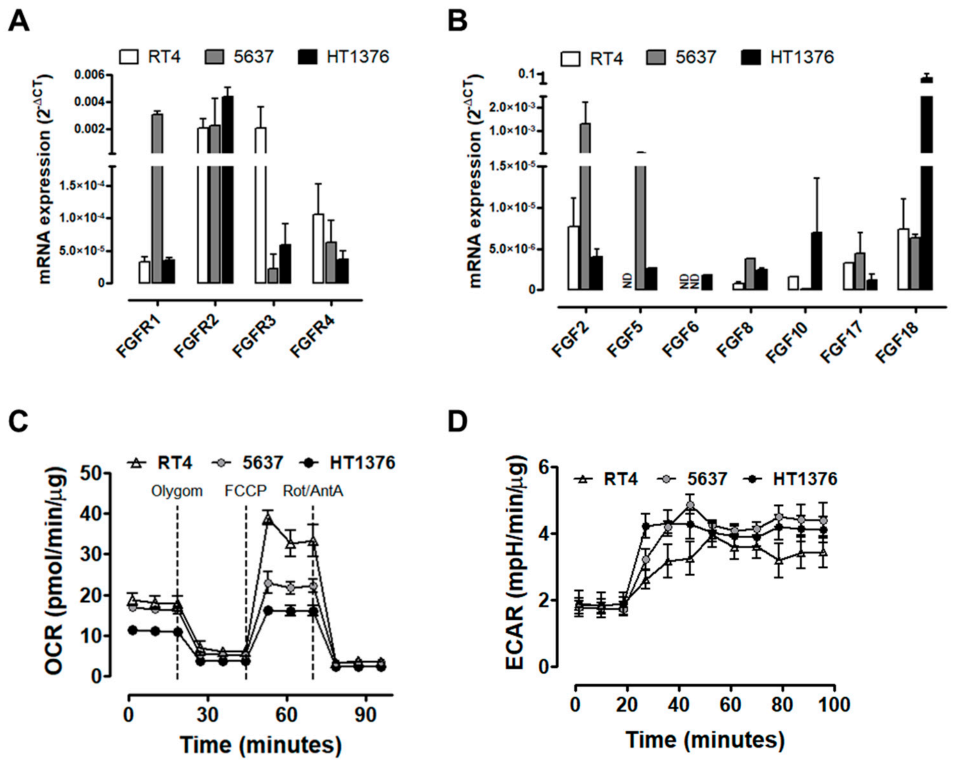

Figure S1. Expression levels by qPCR) of FGFRs (**A**) and FGFs (**B**) in RT4, 5637 and HT1376 cells. The FGF family members not indicated are not detectable in these cells. **C–D**) Seahorse analysis and ECAR levels in RT4, 5637 and HT1376 cells (with reference to Figure 1H in the main text).

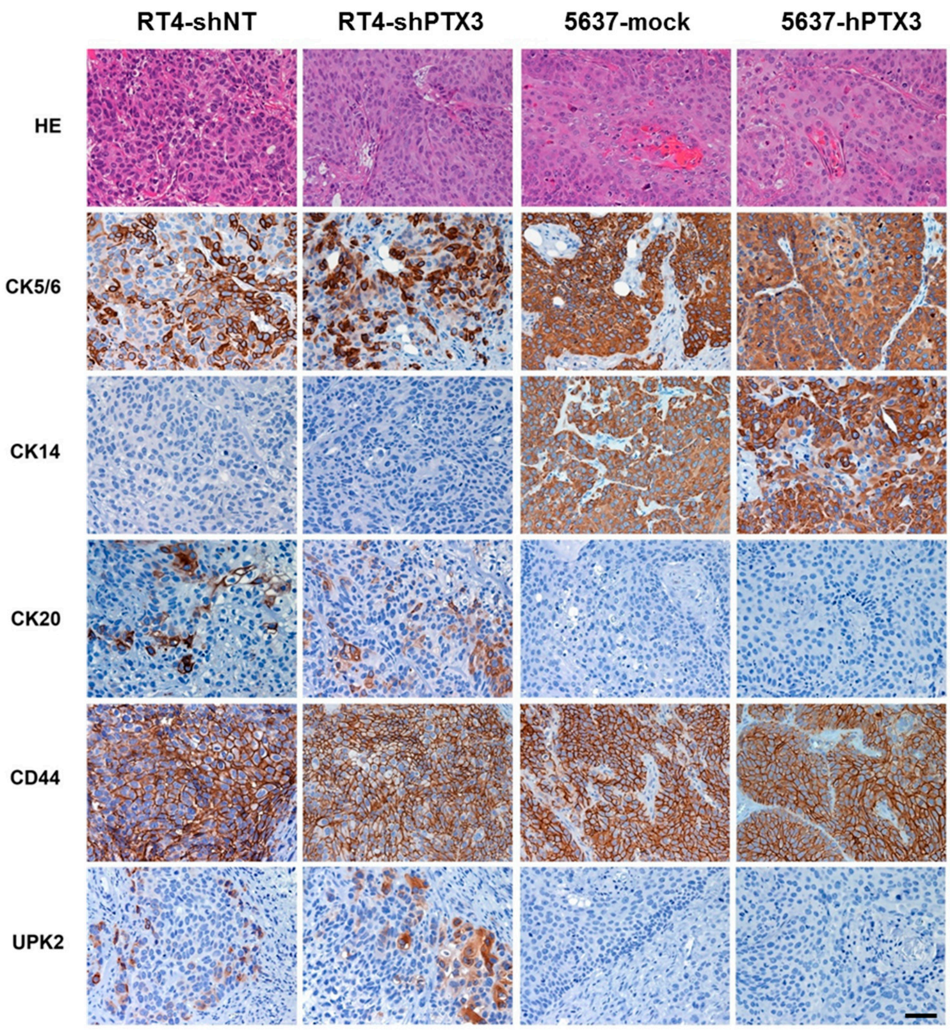

Figure S2. Immunohistochemistry of tumors obtained from RT4-shNT, RT4-shPTX3, 5637-mock, and 5637-hPTX3 cells grafted s.c. in immunedeficient mice. Staining for some of the classical basal (CK14, CK5/6, CD44) and luminal biomarkers (CK20, UPK2) was performed. Scale bar: 50 μm.

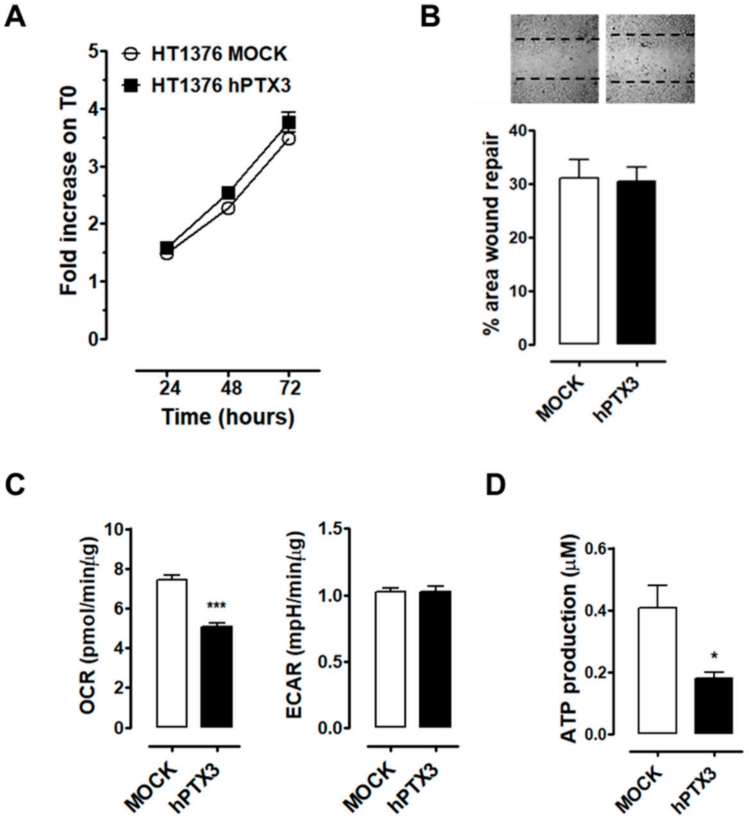

Figure S3. Proliferation (**A**) and wound healing assay (**B**) performed on HT1376-mock and HT1376-PTX3 cells. OCR and EACR measured using Seahorse platform (**C**), and ATP production by HT1376-mock and HT1376-PTX3 cells (**D**). * $p < 0.05$, *** $p < 0.001$.

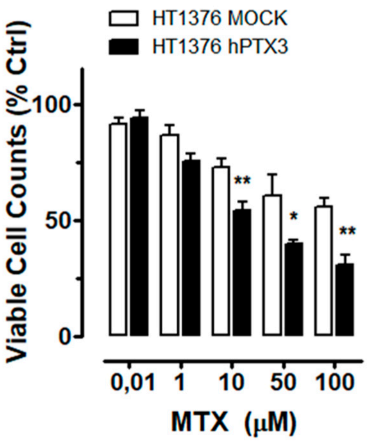

Figure S4. Viable cell counting of HT1376-Mock and HT1376-PTX3 cells treated with increasing concentrations of Methotrexate (MTX). Mean ±SEM; * $p < 0.05$, ** $p < 0.01$.

Supplementary Materials and Methods

Cell culture details

Human RT4, 5637 and HT1376 cells are from American Type Culture Collection (ATCC); murine MB49 cells are a kind gift from Dr. O'Donnell (University of Iowa Hospitals and Clinics, Iowa City, IA, USA). Cells were cultured in different medium (RT4: McCoy's 5A; 5637: RPMI; HT1376 and MB49: DMEM), containing penicillin/streptomycin (100U and 10 mg/mL, respectively) and supplemented with 10% fetal bovine serum (FBS-Gibco, Gaithersburg, MD, USA) and maintained at 37°C with 5% CO_2. 5637, HT1376 and MB49 bladder cells were transfected with a pBABE-Puro vector harboring the full length human PTX3 cDNA (GenBank accession n° X63613) or a pBABE-Puro empty vector (Mock) using FuGENE (Promega, Milan, Italy). RT4 cells were silenced using short-hairpin RNA (shRNA) lentiviral vectors targeting the human PTX3 sequence (TRCN0000436981; Sigma-Aldrich, Milan Italy) and a scrambled/control sequence (SHC002V; Sigma-Aldrich). Cells were maintained at low passage, and tested regularly for Mycoplasma negativity.

Western Blot Analysis

Cells were lysed in NP-40 lysis buffer (1% NP-40, 20 mM Tris-HCl pH 8.0, 137 mM NaCl, 10% glycerol, 2.0 mM EDTA, 1.0 mM sodium orthovanadate and protease inhibitors cocktail) and 30µg loaded on gel. Antibodies: anti-PTX3 antibody from B. Bottazzi (Humanitas Clinical Institute, Milan, Italy), anti-pFGFR, anti-pFRS2 and anti-pERK1/2 antibodies (Cell Signaling), and normalized with an anti-GAPDH antibody (Santa Cruz Biotechnology, Dallas, TX, USA).

PCR Analysis

Total RNA was extracted using TRIzol Reagent accordingly to manufacturer's instructions (Invitrogen, Carlsbad, CA, USA) and 2.0 µg of total RNA were retro-transcribed with MMLV reverse transcriptase using random hexaprimers in a final 20 µL volume. The cDNA was used as template in PCR reactions using specific primers (see **Table S1**).

Western Blot gels relative to Figure 1.

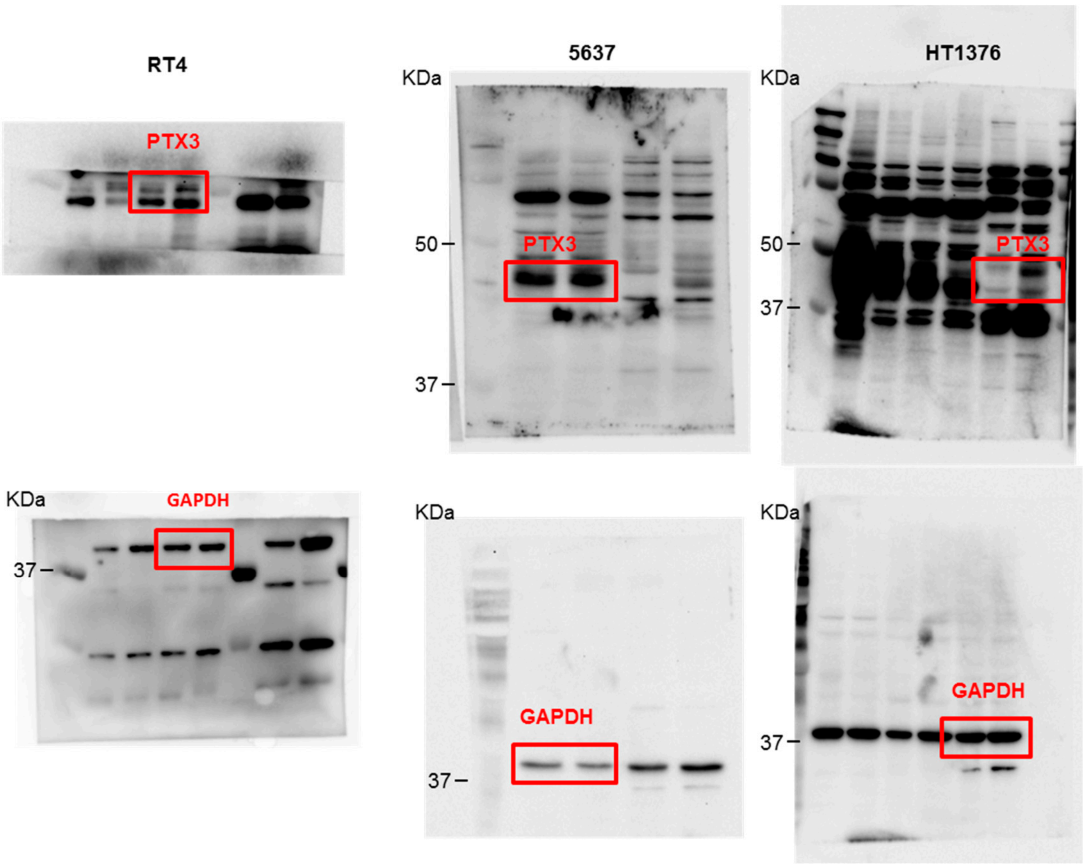

After transfer the membrane was cut and incubated with the indicated Abs and acquired separately with a BioRad ChemiDoc Imaging System.

Western Blot gels relative to Figure 2.

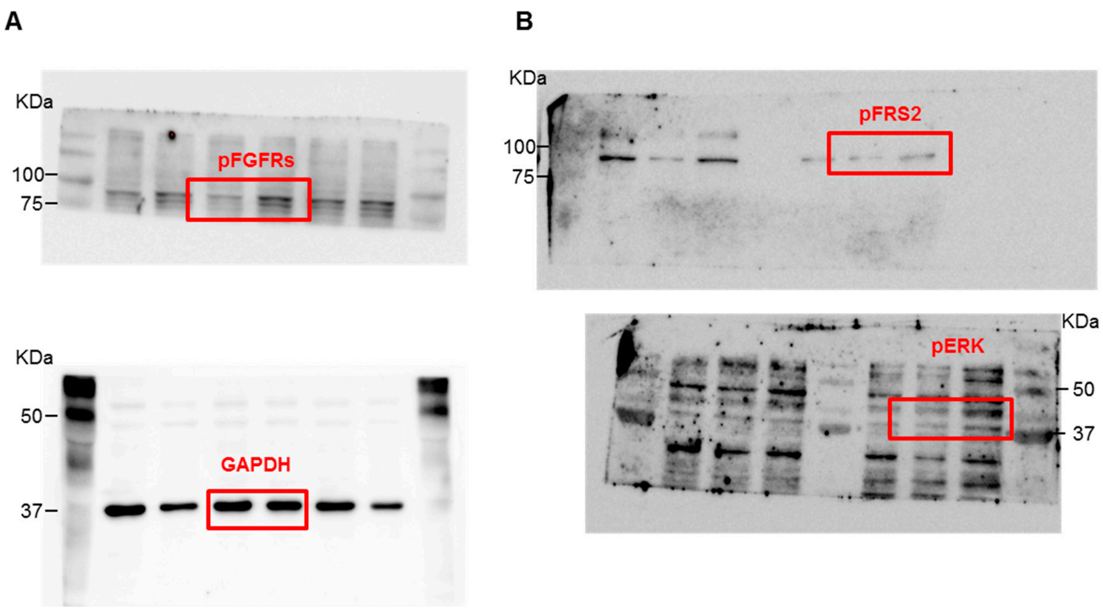

A) After transfer the membrane was cut and incubated with the indicated Abs and acquired separately with a BioRad ChemiDoc Imaging System. **B**) Then, a second aliquot of the same cell extracts was loaded on a different gel and incubated with the indicated Abs.

Western Blot gels relative to Figure 3.

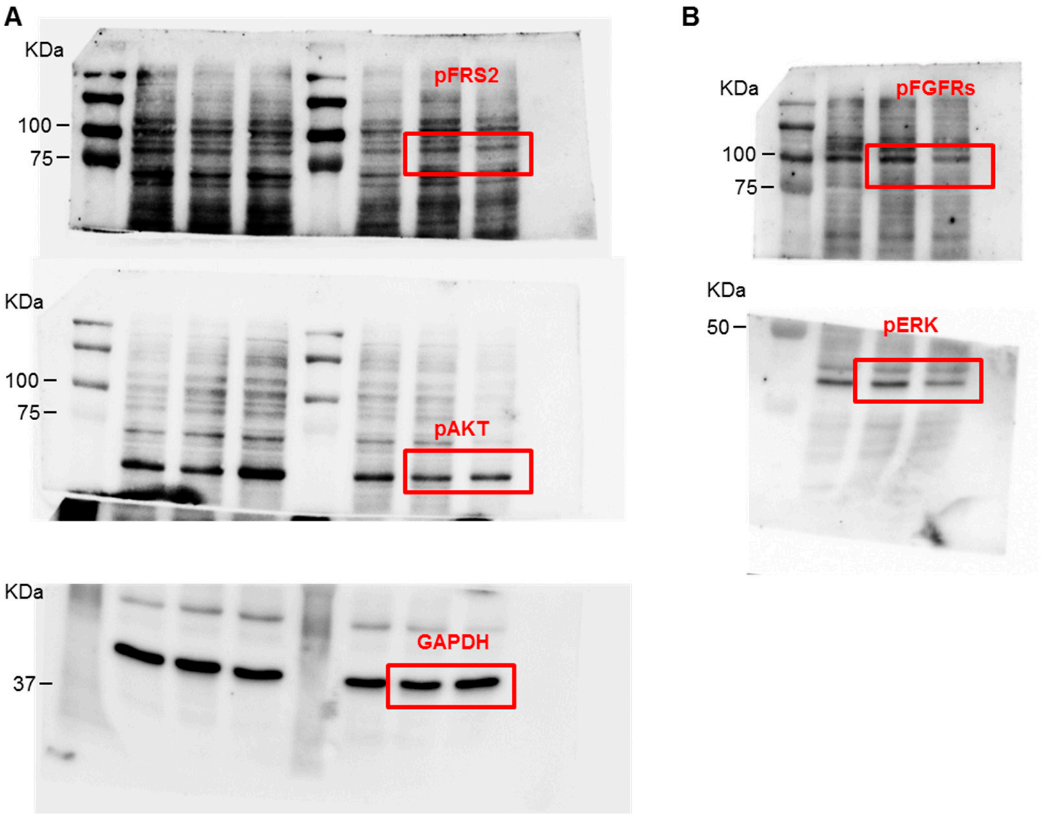

A) After transfer the membrane was cut and incubated with the indicated Abs and acquired separately with a BioRad ChemiDoc Imaging System. **B**) Then, a second aliquot of the same cell extracts was loaded on a different gel and incubated with the indicated Abs.

Western Blot gels relative to Figure 4.

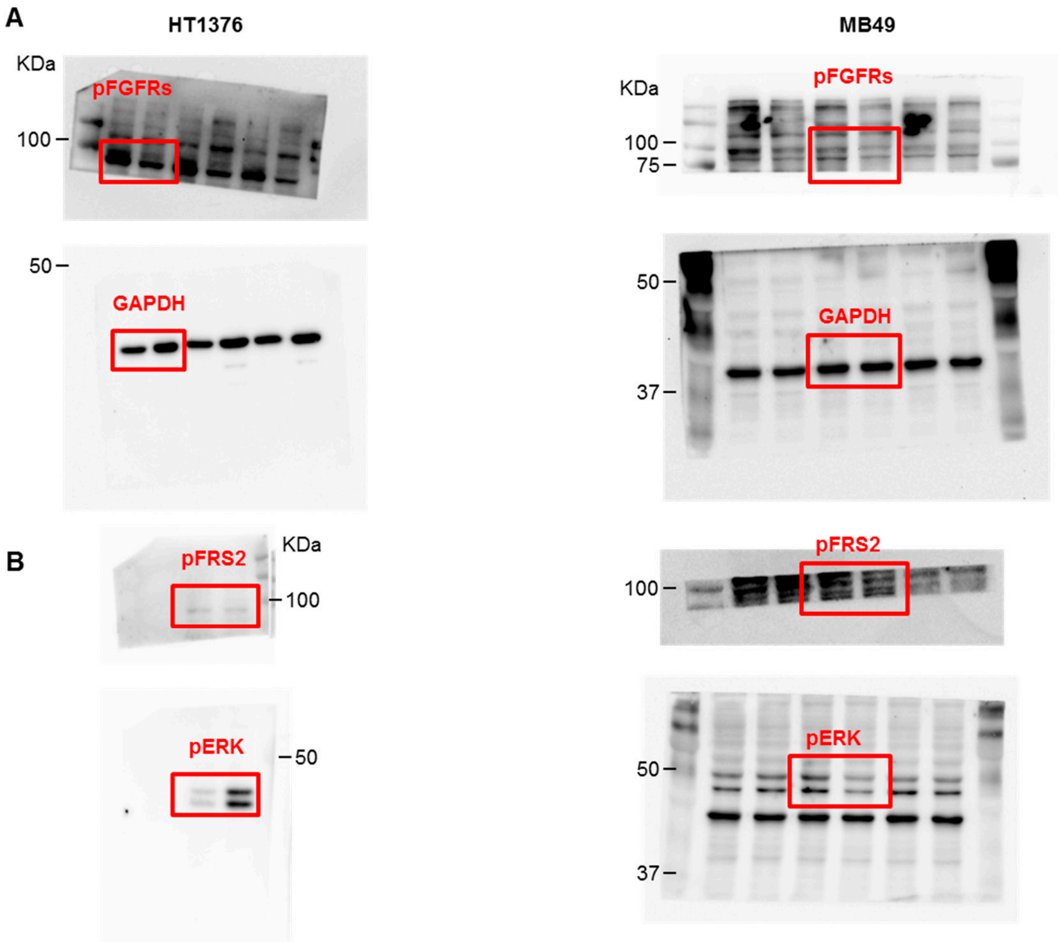

A) After transfer the membrane was cut and incubated with the indicated Abs and acquired separately with a BioRad ChemiDoc Imaging System. **B**) Then, a second aliquot of the same cell extracts was loaded on a different gel and incubated with the indicated Abs.

Western Blot gels relative to Figure 6.

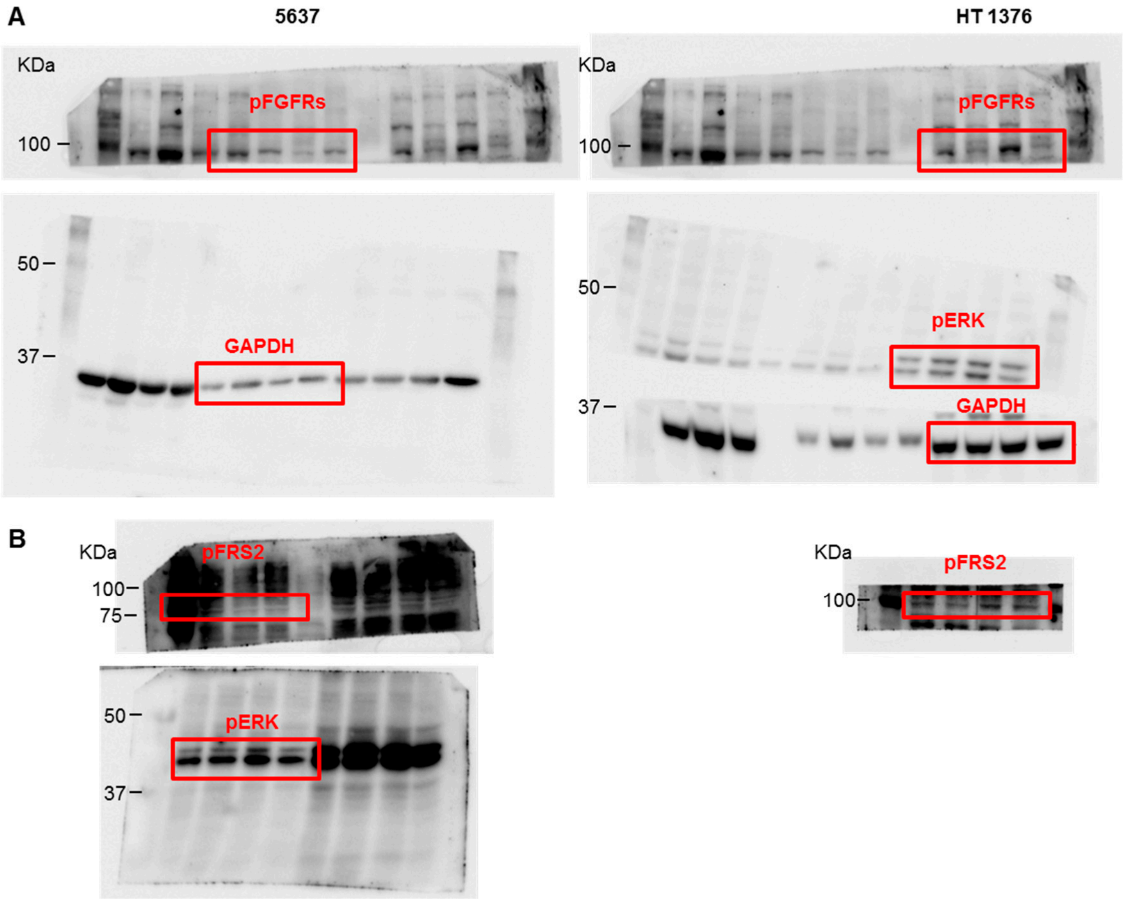

A) After transfer the membrane was cut and incubated with the indicated Abs and acquired separately with a BioRad ChemiDoc Imaging System. **B**) Then, a second aliquot of the same cell extracts was loaded on a different gel and incubated with the indicated Abs.

Figure S5. Western Blot gels relative to Figures 1–4 and 6.
